# Supplementary material for: Modeling glioblastoma heterogeneity as a dynamic network of cell states
Source: Mol Syst Biol. 2021 Sep 16;17(9):e10105. doi: 10.15252/msb.202010105 (PMC8444284; doi:10.15252/msb.202010105)
Supplement: Supplementary file 6 — Source Data for Figure 5 [file MSB-17-e10105-s004.zip › Figure5A_sourcedata/GSEA_3071/hallmarks_stateV.GseaPreranked.1623742546329/HALLMARK_SPERMATOGENESIS.html]

Details for gene set HALLMARK\_SPERMATOGENESIS[GSEA]

|  || Dataset | state43071 |
| Phenotype | NoPhenotypeAvailable |
| Upregulated in class | na\_pos |
| GeneSet | HALLMARK\_SPERMATOGENESIS |
| Enrichment Score (ES) | 0.5438698 |
| Normalized Enrichment Score (NES) | 2.3581097 |
| Nominal p-value | 0.0 |
| FDR q-value | 0.0 |
| FWER p-Value | 0.0 |
Table: GSEA Results Summary

  

Fig 1: Enrichment plot: HALLMARK\_SPERMATOGENESIS      
 Profile of the Running ES Score & Positions of GeneSet Members on the Rank Ordered List

  

| PROBE | GENE SYMBOL | GENE\_TITLE | RANK IN GENE LIST | RANK METRIC SCORE | RUNNING ES | CORE ENRICHMENT || 1 | EZH2 |  |  | 55 | 1.534 | 0.0449 | Yes |
| 2 | CDK1 |  |  | 69 | 1.488 | 0.0962 | Yes |
| 3 | TOPBP1 |  |  | 108 | 1.350 | 0.1377 | Yes |
| 4 | CDKN3 |  |  | 163 | 1.239 | 0.1722 | Yes |
| 5 | NCAPH |  |  | 168 | 1.235 | 0.2160 | Yes |
| 6 | RFC4 |  |  | 171 | 1.227 | 0.2599 | Yes |
| 7 | DBF4 |  |  | 189 | 1.197 | 0.2999 | Yes |
| 8 | BUB1 |  |  | 277 | 1.084 | 0.3225 | Yes |
| 9 | TTK |  |  | 383 | 0.966 | 0.3374 | Yes |
| 10 | SPATA6 |  |  | 404 | 0.946 | 0.3678 | Yes |
| 11 | CNIH2 |  |  | 434 | 0.920 | 0.3955 | Yes |
| 12 | PCSK1N |  |  | 476 | 0.887 | 0.4197 | Yes |
| 13 | PIAS2 |  |  | 504 | 0.862 | 0.4457 | Yes |
| 14 | JAM3 |  |  | 704 | 0.745 | 0.4348 | Yes |
| 15 | AURKA |  |  | 761 | 0.718 | 0.4500 | Yes |
| 16 | HSPA4L |  |  | 793 | 0.703 | 0.4695 | Yes |
| 17 | PARP2 |  |  | 864 | 0.673 | 0.4805 | Yes |
| 18 | STRBP |  |  | 865 | 0.673 | 0.5048 | Yes |
| 19 | NEK2 |  |  | 901 | 0.659 | 0.5219 | Yes |
| 20 | GAD1 |  |  | 947 | 0.641 | 0.5365 | Yes |
| 21 | PEBP1 |  |  | 1026 | 0.614 | 0.5439 | Yes |
| 22 | KIF2C |  |  | 1309 | 0.530 | 0.5094 | No |
| 23 | CCNB2 |  |  | 1373 | 0.513 | 0.5159 | No |
| 24 | DCC |  |  | 1504 | 0.479 | 0.5085 | No |
| 25 | PHF7 |  |  | 1927 | 0.400 | 0.4427 | No |
| 26 | GMCL1 |  |  | 2016 | 0.385 | 0.4399 | No |
| 27 | IP6K1 |  |  | 2025 | 0.384 | 0.4522 | No |
| 28 | CAMK4 |  |  | 2302 | 0.349 | 0.4123 | No |
| 29 | SIRT1 |  |  | 2640 | 0.305 | 0.3592 | No |
| 30 | AGFG1 |  |  | 2695 | 0.300 | 0.3598 | No |
| 31 | VDAC3 |  |  | 2803 | 0.289 | 0.3498 | No |
| 32 | PHKG2 |  |  | 3009 | 0.268 | 0.3205 | No |
| 33 | HSPA2 |  |  | 3149 | 0.255 | 0.3033 | No |
| 34 | NPHP1 |  |  | 3194 | -0.250 | 0.3040 | No |
| 35 | STAM2 |  |  | 3618 | -0.318 | 0.2350 | No |
| 36 | GSTM3 |  |  | 3966 | -0.381 | 0.1827 | No |
| 37 | PSMG1 |  |  | 4573 | -0.574 | 0.0882 | No |
| 38 | ARL4A |  |  | 4593 | -0.582 | 0.1056 | No |
| 39 | NF2 |  |  | 4864 | -0.769 | 0.0820 | No |
Table: GSEA details [plain text format]

  

Fig 2: HALLMARK\_SPERMATOGENESIS: Random ES distribution      
 Gene set null distribution of ES for **HALLMARK\_SPERMATOGENESIS**
